# Brain Resources: How Semantic Cueing Works in Mild Cognitive Impairment due to Alzheimer’s Disease (MCI-AD)

**DOI:** 10.3390/diagnostics11010108

**Published:** 2021-01-12

**Authors:** Andrea Brugnolo, Nicola Girtler, Elisa Doglione, Beatrice Orso, Federico Massa, Maria Isabella Donegani, Matteo Bauckneht, Silvia Morbelli, Dario Arnaldi, Flavio Nobili, Matteo Pardini

**Affiliations:** 1 Department of Neuroscience, Rehabilitation, Ophthalmology, Genetics, and Mother-Child health (DINOGMI), University of Genoa, 16132 Genova, Italy; nicolagirtler@unige.it (N.G.); elisa.doglione@gmail.com (E.D.); beatrice27orso@gmail.com (B.O.); fedemassa88@gmail.com (F.M.); dario.arnaldi@gmail.com (D.A.); flaviomariano.nobili@hsanmartino.it (F.N.); matteo.pardini@unige.it (M.P.); 2 Clinical Psychology Unit, IRCCS Ospedale Policlinico San Martino, 16132 Genoa, Italy; 3 Department of Health Sciences (DISSAL), University of Genoa, 16132 Genoa, Italy; isabella.donegani@gmail.com (M.I.D.); bauckneht@yahoo.com (M.B.); silviadaniela.morbelli@hsanmartino.it (S.M.); 4 Nuclear Medicine Unit, IRCCS Ospedale Policlinico San Martino, 16132 Genoa, Italy; 5 Neurology Clinics, IRCCS Ospedale Policlinico San Martino, 16132 Genoa, Italy

**Keywords:** FCSRT, semantic cue, MCI-AD

## Abstract

Semantic cues in the Free and Cued Selective Reminding Test (FCRST) play a key role in the neuropsychological diagnosis of Amnesic Mild Cognitive Impairment due to Alzheimer’s Disease (MCI-AD); however, the neural bases of their impact of recall abilities are only partially understood. Here, we thus decided to investigate the relationships between brain metabolism and the FCSRT Index of Sensitivity of Cueing (ISC) in patients with MCI-AD and in healthy controls (HC). *Materials:* Thirty MCI-AD patients (age: 74.7 ± 5.7 years; education: 9.6 ± 4.6 years, MMSE score: 24.8 ± 3.3, 23 females) and seventeen HC (age: 66.5 ± 11.1 years; education: 11.53 ± 4.2 years, MMSE score: 28.4 ± 1.14, 10 females) who underwent neuropsychological evaluation and brain F-18 fluorodeoxyglucose Positron Emission Tomography (FDG-PET) were included in the study. *Results:* ISC was able to differentiate HC from MCI-AD subjects as shown by a ROC analysis (AUC of 0.978, effect size Hedges’s g = 2.89). MCI-AD subjects showed significant hypometabolism in posterior cortices, including bilateral inferior Parietal Lobule and Precuneus and Middle Temporal gyrus in the left hemisphere (VOI-1) compared to HC. ISC was positively correlated with brain metabolism in a single cluster (VOI-2) spanning the left prefrontal cortex (superior frontal gyrus) and anterior cingulate cortex (ACC) in the patient group (R^2^ = 0.526, *p* < 0.001), but not in HC. Mean uptake values of VOI-2 did not differ between HC and MCI-AD. The structural connectivity analysis showed that VOI-2 is connected with the temporal pole, the cingulate gyrus and the posterior temporal cortices in the left hemisphere. *Conclusion:* In MCI-AD, the relative preservation of frontal cortex metabolic levels and their correlation with the ISC suggest that the left frontal cortices play a significant role in maintaining a relatively good memory performance despite the presence of posterior hypometabolism in MCI-AD.

## 1. Introduction

Formal assessment of memory is a key step to correctly identify subjects with Mild Cognitive Impairment due to Alzheimer’s Disease (MCI-AD) [1] and to define subjects with subjective memory complaints as well as to stratify asymptomatic subjects at risk for AD [2]. While there are several useful tests to assess memory performance, the Free and Cued Selective Reminding Test (FCSRT) is suggested to be one of the most sensitive tests to evaluate memory in AD by the International Working Group [3] and by the Joint Program for the Neurodegenerative Working Group [4]. Indeed, the FCSRT has been shown to have a greater accuracy than other tests to discriminate MCI-AD from other forms of MCI and to predict the risk of conversion from MCI to AD dementia [5,6,7]. 

Even if the FCRST allows the computation of several indices of immediate and delayed recall, it also makes it possible to evaluate the impact of semantic cues on recall. This easy-to-compute score (i.e., the Index of Sensitivity of Cueing (ISC) is clinically relevant as the presence of recall deficits with a limited effect of semantic cue is highly suggestive of a hippocampal syndrome, which represents a typical presentation of typical AD [3,8,9,10].

Interestingly, the ISC is partially altered in the first clinical stage of AD and it declines with the progression of the disease without suffering by the floor effect even at the moderate stage of the disease [11]. Indeed, it is one of the best indexes to estimate the risk of conversion from MCI to AD [5,7].

So far, the neural bases of ISC are only partially understood. Longitudinal MRI studies in MCI-AD compared with healthy controls suggest that ISC is related to the loss of gray and white matter in the frontal lobe. [6,12]. While these findings seem to be in line with current models of memory [13] and with the psychometric features of ISC in the differential diagnosis between AD and other neurodegenerative conditions, with an earlier and more severe impairment of the frontal lobes [14,15], other functional imaging studies did not show any association between ISC and frontal lobe functionality [16,17]. Whilst these discrepancies may be due to differences in inclusion criteria, to the version of the FCSRT used or to neuroimaging protocols, as well as to the lack of a proper control group of healthy subjects, nevertheless it reduces our confidence in the understanding of the neural bases of ISC and thus of its clinical strength. 

In order to fill this gap, we decided to investigate the neural bases of ISC using a newly validated, robust version of the FCSRT [18] and brain FDG-PET, focusing both on well characterized MCI-AD subjects and matched healthy controls. 

## 2. Materials and Methods

### 2.1. Patients

The study was conducted on thirty consecutive patients with MCI due to AD (MCI-AD) (age: 74.7 ± 5.7 years; education: 9.6 ± 4.6 years, MMSE score at the time of neuropsychological evaluation: 24.8 ± 3.3, 23 females) enrolled in our memory clinic, who underwent a complete diagnostic work-up according to current criteria [1]. This included general and neurological examinations, standardized neuropsychological assessments, brain structural imaging with Magnetic Resonance Imaging (MRI), brain FDG-PET, and, in selected patients, amyloid PET. The diagnosis of probable AD was confirmed by the conversion to AD dementia at subsequent follow-up visits (mean conversion time: 21.0 ± 9.5 months, range: 6–42). For patients and controls (see below), MRI evidence of stroke or of an intracranial space-occupying lesion were considered an exclusion criterion, while white matter hyperintensities, leucoaraiosis, and lacunae did not constitute an exclusion criterion if the Wahlund score was <3 in all regions [19]. Drugs known to interfere with brain metabolism and perfusion were slowly tapered and withdrawn whenever possible before the neuropsychological and FDG-PET examinations. 

### 2.2. Controls

Controls (HC) were seventeen healthy volunteers (age: 66.5 ± 11.1 years; education: 11.53 ± 4.2 years, MMSE score at the time of neuropsychological evaluation: 28.4 ± 1.14, 10 females) who gave their informed consent to participate in the study and who were recruited during university courses dedicated to older adults. All controls were evaluated with a general and a neurological examination as well as with the same standardized neuropsychological test battery used for patients, with normal findings. The Clinical Dementia Rating scale was 0 and the MMSE score was >26. HC subjects underwent brain FDG-PET scan, which was read as normal. After a mean follow-up time of 3 years, all were confirmed to be in healthy condition. 

### 2.3. Neuropsychological Evaluation

The study is focused on the word version of the FCSRT standardized for the Italian population. The test evaluates free and cued immediate and delayed memory, as well as recognition performance. Overall, the test defines the memory profile with a total of six indexes, i.e., IFR (Immediate Free Recall), ITR (Immediate total Recall), ISC (Index of Cue Sensitivity), R (Recognition), DFR (delayed free recall), and DTR (Delayed Total recall). 

The FCSRT is part of the same neuropsychological battery administered to patients and controls. This battery includes the Trail making test (TMT-A and B, with computation of B-A score) to explore visuomotor abilities, divided attention, and attention shifting; the Stroop color-word test for cognitive flexibility and executive functions; the symbol digit test to assess executive functions and working memory; the Corsi’s block design to investigate spatial memory; the digit span (forward) assessing auditory memory span; and the Clock Completion test as a mixed measure of executive functions, visuospatial abilities, and memory; the categorical and phonological verbal fluency test; the figure copying of the mental deterioration battery (simple copy and copy with guiding landmarks) to assess visuoconstructional abilities. References for tests and normative values are listed in a previous paper [20]. 

### 2.4. FDG-PET Acquisition

FDG-PET was performed within two months from the baseline clinical-neuropsychological examination. Subjects fasted for at least six hours. Before radiopharmaceutical injection, blood glucose was checked and was <140 mg/dL in all subjects. After a 10-min rest in a silent and obscured room, with eyes open and ears unplugged, subjects were injected with 185–300 MBq of ^18^F-FDG via a venous cannula, according to the first guidelines of the European Association of Nuclear Medicine (EANM) [21]. They remained in the room for 30 min after the injection and then moved to the PET room, where scanning started approximately 45 min after the injection and lasted another 15 min. Emission scans were acquired in 3-dimensional mode and corrected for attenuation based on CT scan. Images were reconstructed using an ordered subset expectation maximization (OSEM) algorithm. 

### 2.5. Image Analysis

FDG-PET data were subjected to affine and nonlinear spatial normalization into the Talairach, and Tournoux’s space using SPM8 (Wellcome Department of Cognitive Neurology, London, UK) implemented in Matlab 7.5 (Mathworks, Natick, MA, USA). The normalized images were then smoothed with a 8 mm FWHM (Full Width at a Half Maximum) isotropic Gaussian filter and then processed in SPM. In all the analyses, the standard 0.8 gray matter threshold masking as well as the default value of 50 for the grand mean scaling were used. All the default choices of SPM8 were followed with one main exception. To avoid inconsistencies deriving from the use of the default SPM brain H2O template [22], PET scans were normalized using a customized brain FDG PET template, as detailed elsewhere [23]. 

### 2.6. Statistical Analysis 

The main demographic and neuropsychological data were analyzed by SPSS 22.0. Neuropsychological test scores were compared between HC and MCI-AD with the *t*-tests. Moreover, we computed the area under the Receiver Operating Characteristic (ROC) curve (AUC) for each index of the FCSRT. 

To evaluate the magnitude of the differences in the indices of the FCSRT between the two groups, the effect size was calculated using Hedges’s g statistic [24]. According to Cohen, an effect size higher than 0.5 was regarded as medium, and an effect size higher than 0.8 was regarded as large [25].

FDG-PET comparison (unpaired *t*-test) was performed between HC and patients; moreover, a voxel-wise correlation was run between PET brain metabolism and ISC, both in the HC group and in MCI-AD, with age as a nuisance variable. 

We set a height threshold of *p* < 0.001, uncorrected for multiple comparisons at peak level. We considered as significant only clusters containing at least 50 voxels. According to the SPM t-Map showing the statistically significant clusters, we only considered as significant those clusters with a *p*-value adjusted for search volumes which were statistically significant with *p* < 0.05, family-wise corrected (FWE) for multiple comparisons at cluster level. The mean uptake values of the four significantly hypometabolic clusters obtained in the comparison between MCI-AD and HC (VOI-1) were averaged and then normalized on whole brain counts in each subject using MarsBar software (http:/marsbar.sourceforge.net, release 0.44, 17 March 2016). The same procedure was adopted for the cluster identified as significantly correlated between ISC and brain metabolism in MCI-AD patients (VOI-2) (see the results below). These two VOI values were compared between MCI-AD patients and HC by means of the *t*-test.

### 2.7. Structural Connectivity

To evaluate the structural connectivity of the VOI-2 in the patient group, we applied the BCB toolkit [26,27] using as tractography seed the results of the voxel wise analysis of the MCI-AD FDG images. 

This tool is based on an already available average atlas of white matter (WM) bundles distribution obtained in 35 healthy controls and uses tractography data to map the structural connectivity pattern of user-defined regions. Published data on age effects are available and show the very high anatomical spatial macrostructural similarity between decades [26,27]. 

## 3. Results

### 3.1. Demographics and Neuropsychological Tests 

Demographic and neuropsychological variables for the MCI-AD and HC groups are reported in Table 1 and Table 2. Age significantly differed between groups, while education did not (Table 1). Overall MCI-AD performed significantly worse than HC in all neuropsychological tests (Table 1). Concerning the FCSRT, all indexes reached a *p* < 0.001 level of statistical significance (Table 2) and the effect size was higher than 0.8 for all metrics.

The ROC analysis showed an AUC greater than 0.95 for all FCSRT indexes except for the recognition index (0.888). The effect size computed using Hedges’s g statistic exceeded the value of 0.8 for all the FCSRT indexes (see Table 2).

### 3.2. FDG-PET 

**Figure 1:**
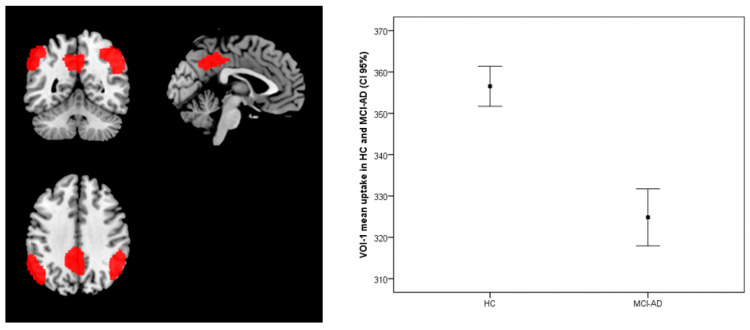
Comparison of brain metabolism between HC and MCI-AD. Significant differences were found with the bilateral inferior parietal lobule, left precuneus and left middle temporal gyrus. Box plot shows the mean uptake values of VOI-1 with a confidence interval (CI) of 95%.

**Figure 2:**
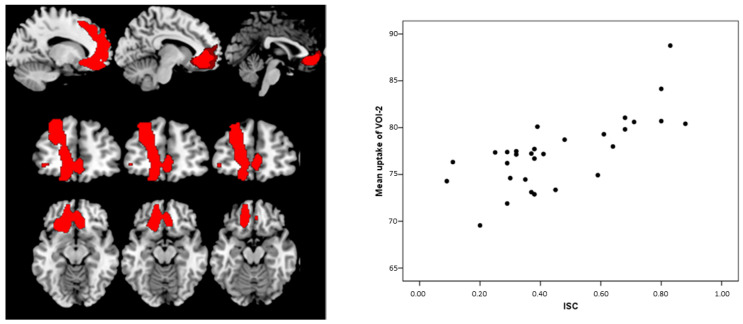
Correlation between Index of Sensitivity of Cueing (ISC) and brain metabolism in MCI-AD. Direct correlation was found in the anterior cingulate and superior frontal gyrus (**left**). Scatterplot of the correlation between VOI-2 metabolism and ISC in MCI-AD. Scatter plot (**right**) shows the correlation between mean uptake values (CI 95%) of the VOI-2 and ISC values. The ISC and relative metabolic values in the left frontal area/ACC are highly correlated (R^2^ = 0.526, *p* < 0.001).

**Figure 3:**
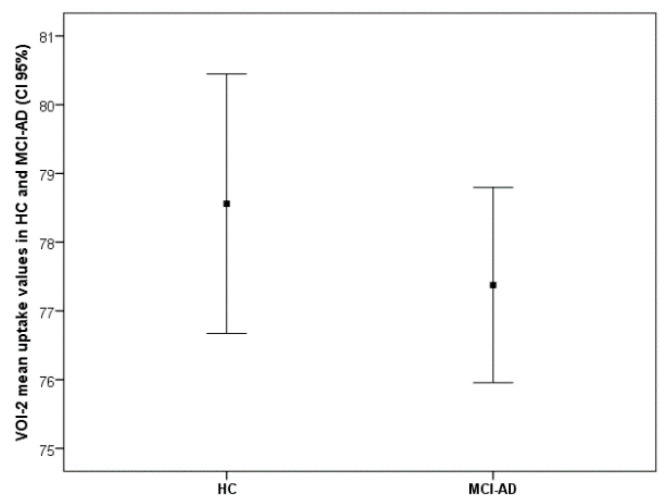
VOI-2 metabolism in HC and MCI-AD. Box plot shows the mean uptake values (CI 95%) of the VOI-2 correlated with ISC. Values of the mean uptake of the VOI-2 do not significantly differ between HC and MCI-AD (*t*-test *p* > 0.05).

As expected, MCI-AD patients showed reduced metabolism in several posterior regions compared with HC, including the bilateral inferior Parietal Lobule and Precuneus and Middle Temporal gyrus in the left hemisphere (VOI-1) (Figure 1, Table 3). The ISC positively correlated with brain metabolism in a single cluster (VOI-2) spanning the left prefrontal cortex (superior frontal gyrus) and anterior cingulate cortex (ACC) only in the patient group (R^2^ = 0.526, *p* < 0.001) (Figure 2, Table 3), while there was no significant correlation in HC. The mean uptake of the VOI-1 obtained by the comparison between MCI-AD and HC significantly differs at the *t*-test (*t*-test *p* < 0.001) (Figure 1), while the mean uptake values of VOI-2 that correlated with ISC did not differ between HC and MCI-AD (*t*-test *p* > 0.05) (Figure 3).

### 3.3. Structural Connectivity

**Figure 4:**
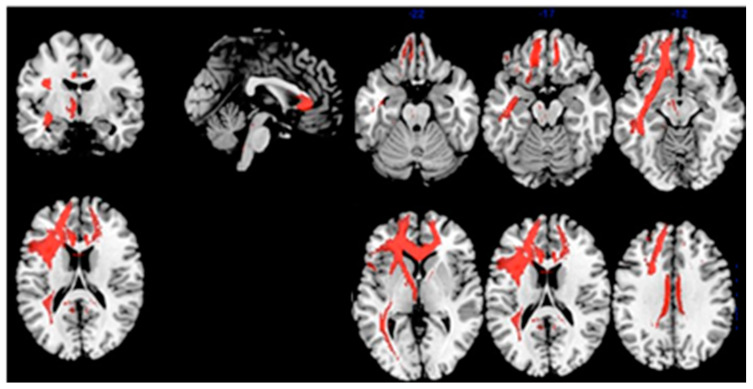
Structural connectivity of theVOI-2. The VOI-2 is connected with the temporal pole (via the uncinated fasciculus), the cingulate gyrus (via the cingulate bundle) and the posterior temporal cortices in the left hemisphere.

The structural connectivity of the VOI-2 is reported in Figure 4, showing the white matter tracts connecting it with the temporal pole (through the uncinate fasciculus), the cingulate gyrus (through the cingulate bundle) and the posterior temporal cortices in the left hemisphere.

## 4. Discussion

In this work, we evaluated the neural bases of ISC using a newly validated, robust version of the FCSRT together with FDG-PET imaging, focusing on a group of well characterized MCI-AD subjects.

We found a positive correlation of ISC with brain metabolism in the VOI-2 that includes the anterior cingulate and the superior frontal gyrus in the left hemisphere in MCI-AD patients. Moreover, using connectivity analyses, we showed that VOI-2 is structurally connected to the temporal pole, the posterior cingulate and the posterior temporal cortices in the left hemisphere, thus presenting rich connections with posterior and anterior cortices involved in memory networks.

The FCSRT evaluates the contribution of frontal areas in memory performance, supporting the retrieval by semantic cue. In healthy subjects, activation of frontal and polar temporal areas in the left hemisphere sustains encoding and recall [28,29]. Moreover, the involvement of frontal cortices in memory performance is also supported by subjects with focal frontal damage in which semantic cues have been shown to dampen the impact of structural damage on memory abilities. The ISC (i.e., the FCSRT index focusing on the impact of semantic cues on memory) seems to be useful to differentiate AD from other neurodegenerative conditions in which memory deficit derives mainly from a malfunctioning of encoding/retrieval strategies (often due to frontal lobe dysfunction) rather than from the reduction in memory trace consolidation [27]. In line with this hypothesis, semantic cues provide more benefits in recall performance in conditions with early frontal damage and relatively preserved mesio-temporal functioning such as fronto-temporal dementia [14,15], rather than AD. In keeping with this interpretation are other longitudinal FCSRT imaging studies showing that ISC values are relatively preserved at the early stage of AD while they decline in subjects with frank dementia, i.e., a stage characterized by significant frontal, as well as temporal atrophy [6,12]. 

The structural connectivity of VOI-2 could explain the differences observed between the results reported in our study and those reported in the literature here below. Indeed, two FDG-PET studies [16,17] did not find a close relationship between frontal metabolic levels and ISC performances, pointing instead to correlation with metabolism in more posterior regions. In the first study, based on the pictorial version of the FCSRT in subjects with MCI, the ISC was reported to correlate with the left posterior cingulate cortex metabolism [16]. Interestingly, the authors pointed out that this area is a hub of a more extensive network devoted to memory functions that also includes frontal regions [16], in keeping with Cabeza et al. (2008) [30]. However, MCI patients were not specified to be precisely MCI due to AD patients, thus likely including different etiologies. In the second study based on the Memento cohort, ISC performance was bilaterally associated with temporal and posterior cortex metabolism [17], while the frontal cortex metabolism correlated only with free recall performance. However, a rather heterogeneous group of subjects with subjective memory complaints, amnesic or non-amnesic single or multiple domain MCI was included. 

Other relevant sources of inhomogeneity between those studies and ours are the use of different versions of the FCSRT, the choice of the PET template used for the normalization [16] and our use of an unbiased, whole brain voxelwise approach—ROI [17]. The use of pictures instead of words as stimuli in memory tasks may have determined some of the observed differences. Indeed, as revealed by a recent study, subjects with posterior lesions in the lateral ventral parietal cortex show a deficit in cued retrieval that is greater for sound–picture association than for word–word pairs [31]. Hence, we can speculate that the FCSRT picture version could be more sensitive to detect the impairment of the posterior network of memory than the FCSRT word version used in this study. Regarding the differences in statistical analysis, the use of a standardized brain parcellation, compared to a voxel-wise approach, does not make it possible to probe the contribution of smaller regions to a given function due to the presence of functionally distinct regions in the same anatomical regions of interest [32]. Indeed, VOI-2 includes some close but distinct anatomo-functional areas, namely the ACC and the superior frontal gyrus (BA 9 and BA 10) that cannot be meaningfully assimilated in an a priori cluster.

As a second result, we found that the metabolic levels of VOI-2 correlated with the ISC in patients but not in HC. Interestingly, VOI-2 FDG uptake did not significantly differ the two groups while the values of ISC were significantly lower in patients compared with HC. 

The relatively preserved brain metabolism of frontal cortices in MCI-AD could play a crucial role in recall facilitation by semantic cues even in subjects with posterior cortices damage. Indeed, according to recent literature, the ACC, the BA 10, and the BA 9 all play key roles in memory functions. The ACC, for example, underpins encoding and associative processes trough synergistic control on the hippocampus and on the fusiform gyrus [33], while BA10 sustains recall processes [34]. The third area of VOI-2 is BA9, whose activation sustains semantic categorization tasks, according to a fMRI study [29]. Considered together, these data suggest that the areas within VOI-2 may play a key role in sustaining memory through several networks that make up for the hippocampus and posterior cortices degeneration. This hypothesis should be further explored using a longitudinal paradigm, as it is well known that in MCI patients, hypometabolism is found earlier in posterior regions rather than in medial temporal lobe and frontal cortices [35,36].

Finally, the mean value of ISC was significantly lower in MCI-AD than in HC, with a high effect size. This observation is in line with the memory profile identified by the FSRT for the early distinguishing of subjects at risk of developing AD, as described by the International Working Group-2 [3]. Indeed, ISC has been considered the most sensitive index to define the different stages of AD, because it progressively declines together with the severity of the disease [11]. In addition, in other studies, the ISC has been revealed to be a good index to predict the conversion from MCI to AD dementia, with a cut-off value of 0.71 indicating a tenfold higher risk of conversion to AD dementia [5].

In conclusion, we explored the neural basis of ISC in MCI-AD. We found a relative preservation of frontal cortex metabolic levels and their correlation with the ISC, suggesting that the left frontal cortices might play a compensatory role despite the presence of posterior hypometabolism in MCI-AD patients.

## Figures and Tables

**Table 1:** Demographic and neuropsychological test values in healthy controls (HC) and in patients with MCI-AD.

	HC (No. 17 ) (Mean ± sd)	MCI-AD ( No. 30) (Mean ± sd)	*t*-Test Values	*p*-Values
Age (y)	66.5 ± 11.1	74.7 ± 5.7	−2.841	0.010
Education (y)	11.5 ± 4.2	9.6 ± 4.6	1.455	0.154
Gender M/F	7/10	7/23	/	/
MMSE	28.4 ± 1.14	24.8 ± 3.3	4.232	0.001
Tmt A	55.9 ± 34.6	87.2 ± 43.5	−2.702	0.010
Tmt B	108.8 ± 93.9	232.7±129.4	- *	-
Symbol Digit	37.8 ± 16.0	20.6 ± 8.2	4.136	0.001
Stroop C	42.0 ± 10.9	31.5 ± 10.3	3.222	0.003
Stroop CW	19.4 ± 9.7	10.4 ± 5.3	3.533	0.002
Digit span	6.1 ± 0.8	5.6 ± 0.9	1.934	0.171
Corsi	4.8 ± 1.1	3.9 ± 0.7	1.433	0.061
Babcock test	13.6 ± 3.9	4.6 ± 2.4	8.532	0.001
CDT	13.9 ± 1.6	11.2 ± 4.4	3.107	0.003
CP	10.1 ± 1.3	8.6 ± 2.0	2.936	0.005
CPE	68.2 ± 2.2	64.6 ± 5.8	3.042	0.004
FVF	36.2 ± 9.7	25.0 ± 9.1	3.832	0.001
SVF	42.9 ± 13.3	25.8 ± 8.1	4.818	0.001
GDS	3.4 ± 3.1	4.6 ± 3.7	−1.207	ns.
ADL	6.0 ± 0	5.8 ± 0.57	1.278	ns.
IADL	8.0 ± 0	6.7 ± 1.5	4.306	0.001
CDR	0 ± 0	0.3 ± 0.2	−7.077	0.001

HC = Controls; MCI-AD = MCI due to Alzheimer’s Disease; TMT-A, TMT-B =Trail Making Test A and B; Stroop C= Stroop Color; Stroop C-W = Stroop Color; CDT = Clock Drawing Test; CP = Constructional Praxis; CPE = Constructional Praxis with Elements; FVF = Fonemic verbal fluency; SVF = Semantic verbal fluency; GDS = Geriatric Depression Scale; ADL Activities of Daily Living; IADL = Instrumental Activity of Daily Living; CDR = Clinical Dementia Rating Scale, ns. = not significant. * TMT-B was not evaluated because the test was discontinued in ten patients out of thirty.

**Table 2:** Values of Free and cued selective reminding test indexes (mean ± sd) in HC and in patients with MCI-AD.

FCSRT	HC (No.17)	MCI-AD (No. 30)	*t*-Test Value	*p* Value	Effect Size	AUC
FCSRT-IFR	25.0 ± 7.2	9.1 ± 6.0	7.731	0.001	1.528	0.951
FCSRT-ITR	46.1 ± 2.2	26.1 ± 10.5	10.024	0.001	2.344	0.988
FCSRT-ISC	0.93 ± 0.08	0.45 ± 0.22	10.764	0.001	2.899	0.978
FCSRT-RP	15.9 ± 0.3	13.4 ± 2.5	5.199	0.001	1.244	0.878
FCSRT-DFR	11.1 ± 2.6	2.7 ± 3.2	9.818	0.001	2.799	0.959
FCSRT-TDR	15.6 ± 0.63	8.5 ± 4.4	0.662	0.001	1.376	0.972

FCSRT = Free and Cue selective Reminding Test; -IFR = Immediate free recall; ITR = Immediate total recall; ISC = Index of sensitive of cueing; RP = Recognition Phase; DFR = Delayed Free Recall; TDR = Total Delayed Recall; AUC = area under the curve.

**Table 3:** SPM results of comparison (height threshold: uncorrected *p* < 0.001) between 17 HC and 30 patients with MCI-AD and of correlation between Index of Sensitivity of Cueing (ISC) and brain metabolism in 30 MCI-AD patients.

Statistic	Cluster Level			Voxel Level			
	Cluster Extent	FWE-Corr. *p* Value	Cortical Region	Z Score of Maximum	Talairach Coordinates	Cortical Region	BA
Comparison: Brain metabolism HC vs. MCI-AD	2191	0.000	L Parietal	5.47	−48, −68, 52	Inf. Parietal Lobule	39
	2189	0.000	R Parietal	5.05	48, −54, 44	Inf. Parietal Lobule	40
	1458	0.000	L Parietal	4.85	−12, −46, 38	Precuneus	31
	1295	0.001	L Temporal	4.22	−72, −26, 4	Middle Temp gy.	21
Correlation: Index of Sensitivity of Cueing (ISC)–brain metabolism in MCI-AD	2481	0.002	L Frontal	4.96	−14, 44, 0	Anterior Cingulate	32
			L Frontal	4.45	−22, 44, 28	Sup. Front. Gy.	9
			L Frontal	4.38	−18, 52, 18	Sup. Front. Gy.	10

FWE = Family Wise Error; Brodmann Area = BA.

## Data Availability

The data presented in this study are available on request from the corresponding author in anonymized form. The data are not publicly available due to lack of consent to public dissemination by the enrolled patients.
